# A sex-based analysis of complete blood count features during acute, untreated Lyme disease

**DOI:** 10.3389/fmed.2024.1454858

**Published:** 2024-10-28

**Authors:** Alison W. Rebman, Ting Yang, Jonathan M. Zenilman, Mark J. Soloski, John N. Aucott

**Affiliations:** ^1^Lyme Disease Research Center, Division of Rheumatology, Department of Medicine, Johns Hopkins University School of Medicine, Baltimore, MD, United States; ^2^Division of Infectious Diseases, Johns Hopkins University School of Medicine, Baltimore, MD, United States

**Keywords:** Lyme disease, complete blood count, lymphocytes, leukocytes, diagnosis, sex-based differences

## Abstract

**Introduction:**

Although lymphopenia has been described in acute Lyme disease (LD), the complete blood count (CBC) has not been comprehensively examined, nor have sex-based analyses been conducted. We analyzed CBC values and identified sex-based trends among patients with early LD by comparing both to controls without a history of LD and to patients’ pre-morbid values.

**Methods:**

We enrolled participants from the Mid-Atlantic US with diagnostic erythema migrans and controls with no history of LD. CBC results were obtained, and patient information was recorded using standardized instruments. We also calculated a neutrophil-to-lymphocyte ratio (NLR). We used linear regression to test that CBC results would differ (a) between antibiotic-naive patients with early LD and controls and (b) by measures of acute disease severity. We also performed stratified analyses to assess sex-based differences.

**Results:**

In total, 236 antibiotic-naive patients with early LD had significantly lower lymphocytes (*β* = −0.34, *p* < 0.001) and significantly higher monocytes (*β* = 0.09, *p* = 0.002) and NLRs (*β* = 0.99, *p* < 0.001) than 61 controls in adjusted analyses. Lymphocytes, monocytes, and NLRs also changed significantly from pre-morbid to acute LD (*p* < 0.001 for all). Only the NLR was consistently significantly associated with disease severity. A higher proportion of male patients with early LD had acute lymphopenia than female patients with early LD (31.93% vs. 19.66%, *p* = 0.03); this difference was not present among controls.

**Conclusion:**

The presence of lymphopenia and the absence of an elevated total white blood cell count make LD an important diagnostic consideration in patients presenting with undiagnosed infectious syndromes in endemic regions. This may be especially true for male patients.

## Introduction

Lyme disease (LD) is a tick-borne infection caused by the bacterium *B. burgdorferi* (1). The number of cases of Lyme and other tick-borne diseases has increased in recent decades across areas of the Northern Hemisphere, and it is the most common vector-borne disease in the United States (2). The diagnosis of early LD relies primarily on physician history and physical examination, which can be challenging as the diagnostic erythema migrans (EM) rash may be hard to identify, especially in people of color (3). At the same time, laboratory diagnosis is limited by the low sensitivity of serology and blood PCR during the first weeks of infection, and direct detection of the infection by culture is currently clinically unavailable (4).

Changes in complete blood cell (CBC) counts during acute *B. burgdorferi* infection have been previously described (5). While several studies have noted lymphopenia, it is seldom mentioned that the neutrophilia seen in early LD is very mild and rarely causes an overall elevated total white blood cell (WBC) count (5). The current Infectious Diseases Society of America (IDSA)’s guidelines also note the possibility for lymphopenia in early LD and suggest that thrombocytopenia, a depressed total WBC count, neutropenia, or anemia may indicate the presence of tick-borne infections, such as anaplasmosis or babesiosis (6). Such descriptions often rely on studies without internal or external control comparisons or on individual clinical experience. Although the neutrophil-to-lymphocyte ratio (NLR) has been recently identified as a biomarker of interest in several infectious diseases, it has not been explored in early LD (7, 8).

Furthermore, prior studies have not controlled for potential demographic differences, such as age, sex, or race/ethnicity, in investigating clinical CBC values during early LD (9–11). In this study, we performed a detailed examination of the CBC presentation of early LD in a large sample of well-characterized patients from the Mid-Atlantic US. We compared patients’ CBC values both to controls without a history of LD and to their pre-morbid values. Importantly, we also examined whether the changes we observed varied by antibiotic exposure and/or patient sex, as sex-and gender-based differences are known to occur across many infectious and non-infectious diseases (12).

The role of the CBC in the clinical diagnosis of early LD may not be known broadly by general practitioners who see patients with acute infectious diseases. Therefore, we sought to contextualize the relevance of our findings for both clinical care and advancing an immunologic understanding of *B. burgdorferi* infection.

## Materials and methods

### Study participants

Adult patients (≥18 years) with early LD, and controls without a clinical history of LD, self-referred or were recruited from primary or urgent care centers and enrolled in a longitudinal cohort study from 2008 to 2023 (13). The Johns Hopkins University School of Medicine Institutional Review Board approved this study, and no study activities were performed prior to the patient providing written consent.

Participants with LD were required to have a visible EM rash of ≥5 cm in diameter diagnosed by a healthcare provider and to have been ill for less than 3 months. They were either antibiotic-naive or had recently begun treatment at the initial study visit (V1); hours since treatment initiation at the time of the visit were recorded. Control participants were recruited from similar care settings as cases through community recruitment, as well as through online and flyer-based advertising. Controls were excluded for a clinical history of prior LD and were required to have a negative two-tier LD serology performed at the time of enrollment. All case and control participants were excluded for any of the following: pregnancy, autoimmune disease, hepatitis B or C, HIV, splenectomy, major psychiatric disease, or cancer/malignancy in the past 2 years.

### Variables of interest

Self-reported gender was obtained for all participants. Additional self-report of sex assigned at birth was added later in data collection and was therefore incomplete. Two participants disclosed transgender and/or non-binary status; for these analyses, they were categorized as their sex assigned at birth.

We analyzed select CBC results (total WBC, lymphocytes, neutrophils, monocytes, platelets, and hematocrit) obtained at V1 from participants with early LD and controls. Absolute values (10^3^ cells/μL) and not percentages were used in all analyses for all results, with the exception of hematocrit. We calculated an NLR by dividing the neutrophil by the lymphocyte counts.

For a subset of patients with LD who had pre-LD CBC values accessible in their medical record, we obtained their consent to review their record and abstracted results from the closest routine, healthy, primary care visit (V0) months to years prior to their LD onset, resulting in paired values for these patients. All CBC tests included in these analyses were performed by one of two CLIA-approved clinical laboratories (88.08 and 11.92% performed by each). Variability in normal reference ranges reported across these two laboratories was accounted for in analyses. For both laboratories, normal reference ranges for hematocrit varied by patient sex, which was taken into account.

We also explored the relationship between CBC results and specific clinical variables of interest. Evidence of early disseminated disease was defined as those patients with multiple EM lesions and/or VII nerve palsy present. Serologic status was determined at V1 using standard two-tier testing (ELISA and Western blot) from a single laboratory, taking illness duration into account (14). EM size (cm^2^) was determined by multiplying two perpendicular measurements. Systemic corticosteroid (e.g., prednisone, prednisolone, and methylprednisolone) exposure in the past 3 months was captured by participant self-report.

### Statistical methods

In unadjusted analyses, we performed chi-square or Fisher’s exact tests for categorical variables and Wilcoxon rank-sum or Spearman’s rank correlation tests for continuous variables. For the subset of patients with CBC results at both V0 and V1, we summarized and directly compared values before and after LD using paired sample *t*-tests. Sign tests were then used to test whether the median percentage change from V0 to V1 differed significantly from 0. All tests were two-sided, and a *p*-value of less than 0.05 was considered to be statistically significant.

To test the hypothesis that CBC results would differ between patients with LD and controls, we generated univariate linear regression models with each CBC result as the outcome, group as the primary independent variable of interest, and evaluated age, sex, laboratory, corticosteroids, race (trichotomized as Black, White, and all other racial groups), and Hispanic ethnicity as potential confounders. Variables identified as significant (*p* < 0.15) were then included in the final adjusted models. A robust M-estimator was used to deal with potential assumption breaches such as non-normal responses and heteroscedasticity. Similar models were also run only among patients with LD to examine the effects of early disseminated disease, serologic status, and EM size on CBC results. For any CBC results in which sex was identified as a significant independent predictor, we performed stratified analyses among patients with early LD and controls to determine whether the sex differences we observed were consistent.

All analyses were performed using SAS (version 9.4, SAS Institute Inc., Cary, NC, United States) or R (version 4.3.1, R Foundation for Statistical Computing, Vienna, Austria), and graphs were generated using GraphPad Prism (version 10.0.2, La Jolla, CA, United States).

## Results

### Participant characteristics

A total of 448 participants (387 patients with LD and 61 controls) were enrolled. One LD patient (0.22%) was excluded for confirmed, concurrent infection with human granulocytic anaplasmosis (HGA), another tick-borne infection. Overall, 15 LD patients (3.35%) lacked at least one V1 CBC result and were also excluded, leaving a final sample of 371 patients with EM and 61 controls (Table 1). A total of 236 (63.61%) of the patients with LD were antibiotic-naive at V1, while 135 (36.39%) were antibiotic-exposed (mean hours since starting treatment: 45.91 ± 35.34, range: 1–167).

**Table 1:** Clinical, demographic, and laboratory characteristics of patients with early Lyme disease and controls without a history of Lyme disease.

	Early Lyme disease with erythema migrans			Controls *N* = 61
	All cases *N* = 371	Antibiotic-naive *N* = 236	Antibiotic-exposed *N* = 135	
Age	52 (38–63) [18–84]	53 (40–62) [20–84]	49 (37–65) [18–83]	50 (35–63) [20–80]
Female	177 (47.71%)	117 (49.58%)	60 (44.44%)	34 (55.74%)
Race/ethnicity				
White	352 (94.88%)	224 (94.92%)	128 (94.81%)	52 (85.25%)
Black	3 (0.81%)	1 (0.42%)	2 (1.48%)	5 (8.20%)
Asian	6 (1.62%)	3 (1.27%)	3 (2.22%)	1 (1.64%)
Other or >1 race	10 (2.70%)	8 (3.39%)	2 (1.48%)	3 (4.92%)
Hispanic	4 (1.08%)	3 (1.27%)	1 (0.74%)	8 (13.11%)
Clinical characteristics				
Early disseminated disease^a^	112 (30.19%)	72 (30.51%)	40 (29.63%)	N/A
Two-tier positive at V1^b^	105/368 (28.53%)	62/233 (26.61%)	43 (31.85%)	0 (0.00%)
Runny nose, sore throat, or cough in the past 10 days^c^	76/345 (22.03%)	47/211 (22.27%)	29/134 (21.64%)	12/58 (20.69%)
Recent systemic corticosteroid use	11 (2.96%)	6 (2.54%)	5 (3.70%)	0 (0.00%)
Total white blood cells^d^				
Total count (10^3^ cells/μL)	6.60 ± 2.21	6.69 ± 2.35	6.45 ± 1.96	6.00 ± 1.51
Abnormal low (%)	21 (5.66%)	15 (6.36%)	6 (4.44%)	8 (13.11%)
Abnormal high (%)	14 (3.77%)	10 (4.24%)	4 (2.96%)	0 (0.00%)
Lymphocytes				
Total count (10^3^ cells/μL)	1.52 ± 0.60	1.45 ± 0.55	1.65 ± 0.66	1.81 ± 0.51
Abnormal low (%)	87 (23.45%)	61 (25.85%)	26 (19.26%)	2 (3.28%)
Abnormal high (%)	0 (0.00%)	0 (0.00%)	0 (0.00%)	0 (0.00%)
Neutrophils				
Total count (10^3^ cells/μL)	4.28 ± 2.00	4.43 ± 2.11	4.02 ± 1.78	3.55 ± 1.15
Abnormal low (%)	4 (1.08%)	2 (0.85%)	2 (1.48%)	3 (4.92%)
Abnormal high (%)	19 (5.12%)	14 (5.93%)	5 (3.70%)	0 (0.00%)
Monocytes				
Total count (10^3^ cells/μL)	0.61 ± 0.23	0.62 ± 0.24	0.60 ± 0.22	0.49 ± 0.14
Abnormal low (%)	0 (0.00%)	0 (0.00%)	0 (0.00%)	0 (0.00)
Abnormal high (%)	11 (2.96%)	6 (2.54%)	5 (3.70%)	0 (0.0%)
Platelets				
Total count (10^3^ cells/μL)	237.47 ± 69.13	235.83 ± 72.55	240.33 ± 62.87	243.34 ± 59.64
Abnormal low (%)	7 (1.89%)	4 (1.69%)	3 (2.22%)	2 (3.28%)
Abnormal high (%)	3 (0.81%)	1 (0.42%)	2 (1.48%)	0 (0.00%)
Hematocrit				
Total (%)	41.20 ± 3.11	41.33 ± 3.21	40.98 ± 2.91	41.06 ± 3.17
Abnormal low (%)	43 (11.59%)	26 (11.02%)	17 (12.59%)	4 (6.56%)
Abnormal high (%)	3 (0.81%)	3 (1.27%)	0 (0.00%)	1 (1.64%)
Neutrophil-to-lymphocyte ratio^e^
Ratio	3.35 ± 2.41	3.49 ± 2.20	3.10 ± 2.73	2.12 ± 1.07

a Defined as multiple erythema migrans and/or new-onset VII nerve palsy. Among all cases (*n* = 112), 108 (96.43%) had multiple erythema migrans, 3 (2.68%) had multiple erythema migrans and VII nerve palsy, and 1 (0.89%) had single erythema migrans and VII nerve palsy.

b Three antibiotic-naive patients with Lyme disease were missing complete serostatus information at V1. An additional 13.71% (48/350 with complete data) were positive on two-tier testing at a convalescent study visit 3 weeks later following antibiotic treatment.

c Twenty-five antibiotic-naive cases, 1 antibiotic-exposed case, and 3 controls were missing this information at V1.

d Laboratory-defined normal ranges are as follows: Laboratory 1: total white blood cell count: 4.0–11.0 × 10^3^ cells/μL, lymphocytes: 1.1–4.8 × 10^3^ cells/μL, neutrophils: 1.5–7.8 × 10^3^ cells/μL, monocytes: 0.10–1.20 × 10^3^ cells/μL, platelets: 130–450 × 10^3^ cells/μL, hematocrit: male patients: 40–50% and female patients: 35–46%. Laboratory 2: total white blood cell count: 3.8–10.8 × 10^3^ cells/μL, lymphocyte: 0.853.90 × 10^3^ cells/μL, neutrophil: 1.5–7.8 × 10^3^ cells/μL, monocyte: 0.20–0.95 × 10^3^ cells/μL, platelet: 140–400 × 10^3^ cells/μL, hematocrit: male patients: 38.5–50% and female patients: 35–45%.

e Normal ranges have not been adopted across disease states for neutrophil-to-lymphocyte ratios; therefore, the % abnormal values are not included for this marker.

There were no statistically significant differences between all cases and controls by age, sex, laboratory performing the CBC, or recent corticosteroid use (*p* = 0.37, *p* = 0.25, *p* = 0.40, and *p* = 0.35, respectively). However, the proportion of participants self-identifying as Black (*p* = 0.002), White (*p* = 0.01), and Hispanic (*p* < 0.001) was different by group.

The mean CBC values and the proportion above and below laboratory-provided reference ranges by group are shown in Table 1. Descriptively, in comparison with controls, a higher proportion of patients with early LD had abnormally low lymphocytes and abnormally high neutrophils and monocytes.

### Association with antibiotic exposure

As these trends were generally more marked among antibiotic-naive patients, we first examined the effect of antibiotic initiation on CBC results. When tested statistically, we found differences between naïve and exposed patients in lymphocyte (*p* = 0.001) and neutrophil (*p* = 0.03) counts. Furthermore, among antibiotic-exposed patients, increased time since treatment initiation was weakly negatively significantly correlated with WBC (Spearman’s correlation co-efficient: −0.27, *p* = 0.001), neutrophils (−0.39, *p* < 0.001), and monocytes (−0.34, *p* < 0.001) and weakly positively significantly correlated with lymphocytes (0.21, *p* = 0.01). As a result, subsequent analyses focused on antibiotic-naive patients with LD only.

### Patients with Lyme disease compared to controls

We then tested the hypothesis that CBC results would differ between antibiotic-naive patients with LD and controls through multivariate analysis (Table 2). After controlling for age, sex, laboratory, corticosteroids, race, and Hispanic ethnicity, we found that patients with LD had significantly lower lymphocytes and significantly higher neutrophils and monocytes (*p* < 0.005 for each) compared to controls. The NLR, derived from both lymphocyte and neutrophil counts, was also highly significantly different by group (*p* < 0.001). Age, sex, race, and corticosteroids were also found to be independently significantly associated with specific CBC values.

**Table 2:** Unadjusted and adjusted linear regression models to predict CBC test result values, with group (antibiotic-naive patients with early Lyme disease vs. controls) as the primary independent variable of interest and the following included as potential confounders: age, sex, laboratory, race (Black vs. other, and White vs. other), Hispanic ethnicity, and corticosteroids. Only variables identified as significant (*p* < 0.15) in univariate analyses were included in a final adjusted model and presented here.

Outcome	Predictors	Unadjusted		Adjusted	
		Co-efficient (95% CI)	*p*-value	Co-efficient (95% CI)	*p*-value
White blood cell count	Lyme group	0.45 [−0.06, 0.97]	0.08	0.45 [−0.06, 0.97]	0.08
Lymphocyte count	Lyme group	−0.38 [−0.53, −0.24]	<0.001	−0.34 [−0.49, −0.19]	<0.001
	Age (10 years)	−0.04 [−0.08, 0.00]	0.05	−0.04 [−0.08, −0.01]	0.03
	Female	0.19 [0.07, 0.31]	0.003	0.19 [0.07, 0.30]	0.002
	Black (vs. other)	0.40 [−0.11, 0.91]	0.12	0.13 [−0.29, 0.55]	0.55
	Hispanic	0.25 [−0.08, 0.58]	0.13	−0.11 [−0.42, 0.21]	0.51
Neutrophil count	Lyme group	0.61 [0.20, 1.02]	0.004	0.61 [0.20, 1.02]	0.004
	Laboratory	0.48 [−0.07, 1.04]	0.09	0.48 [−0.07, 1.03]	0.09
Monocyte count	Lyme group	0.11 [0.06, 0.16]	<0.001	0.09 [0.03, 0.15]	0.002
	Female	−0.08 [−0.13, −0.04]	<0.001	−0.08 [−0.12, −0.03]	<0.001
	White (vs. other)	0.08 [−0.02, 0.18]	0.14	0.08 [−0.01, 0.17]	0.07
	Hispanic	−0.14 [−0.26, −0.02]	0.02	−0.07 [−0.19, 0.05]	0.23
	Corticosteroids	0.22 [0.06, 0.38]	0.006	0.20 [0.04, 0.35]	0.01
Platelet count	Lyme group	−11.88 [−31.20, 7.44]	0.23	−5.17 [−23.90, 13.56]	0.59
	Female	40.59 [25.58, 55.61]	<0.001	39.45 [24.69, 54.22]	<0.001
	Black (vs. other)	82.83 [19.53, 146.13]	0.01	55.83 [2.06, 109.60]	0.04
Hematocrit count	Lyme group	0.18 [−0.74, 1.10]	0.69	−0.13 [−0.94, 0.68]	0.75
	Age (10 years)	−0.19 [−0.44, 0.05]	0.12	0.01 [−0.21, 0.22]	0.96
	Female patients	−3.20 [−3.82, −2.57]	<0.001	−3.16 [−3.81, −2.51]	<0.001
	Hispanic	−2.02 [−4.01, −0.02]	0.05	−1.33 [−3.07, 0.42]	0.14
Neutrophil-to-lymphocyte ratio	Lyme group	1.12 [0.71, 1.54]	<0.001	0.99 [0.55, 1.42]	<0.001
	Age (10 years)	0.10 [−0.01, 0.22]	0.08	0.08 [−0.03, 0.19]	0.17
	Female patients	−0.43 [−0.78, −0.09]	0.01	−0.38 [−0.72, −0.04]	0.03
	Laboratory	0.44 [−0.16, 1.03]	0.15	0.45 [−0.11, 1.00]	0.11
	Black (vs. other)	−1.14 [−2.61, 0.34]	0.13	−0.72 [−1.94, 0.50]	0.25
	Hispanic	−0.90 [−1.85, 0.05]	0.06	−0.15 [−1.07, 0.76]	0.74

### Changes from pre-morbid values

**Figure 1:**
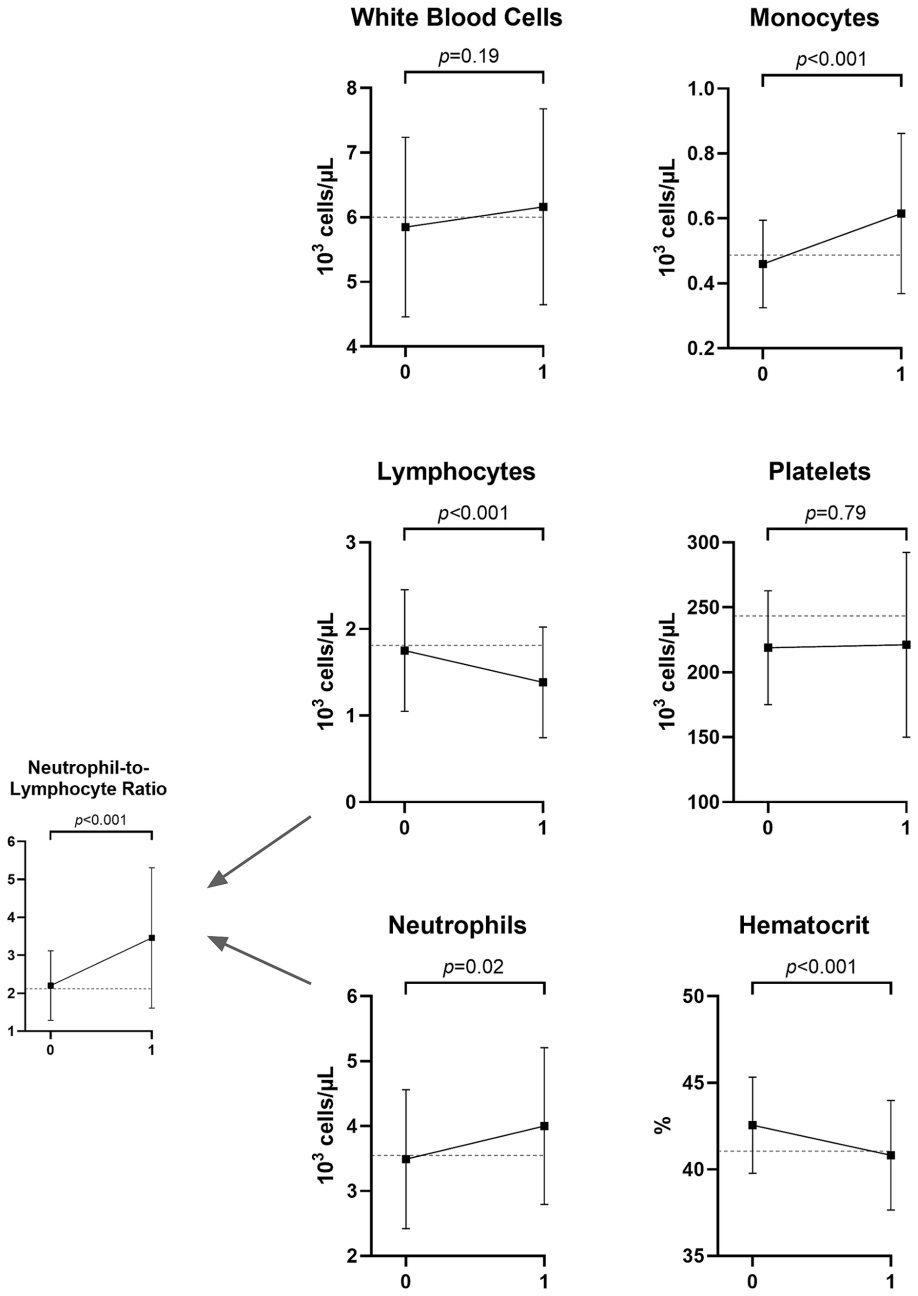
Mean and standard deviation of CBC values months to years before (V0) and at the time of Lyme disease diagnosis (V1) among 46 patients who were antibiotic-naive at V1. The dotted line displays the mean value for a sample of 61 controls without Lyme disease. The *p*-values represent paired sample *t*-tests comparing patients with Lyme disease at the two time points.

We then tested the change pattern in CBC results from patients’ pre-morbid (V0) to early LD (V1) visits. Among antibiotic-naive patients, 46 had paired V0/V1 data, with a median time from V0 to V1 of 12.19 months (IQR: 6.44–24.35, range 2.99–128.40). Figure 1 depicts the change in CBC results from V0 to V1 in this sample of patients. The median percentage change from V0 to V1 for each was as follows: total WBC: 3.89% (*p*-value for significance of difference from 0 = 0.44), lymphocytes: −14.34% (*p* < 0.001), neutrophils: 16.52% (*p* = 0.10), monocytes: 24.47% (*p* < 0.001), platelets: −4.65% (*p* = 0.30), hematocrit: −4.30% (*p* < 0.001), and NLR: 45.32% (*p* < 0.001).

### Relationship to clinical disease severity

After controlling for age, sex, laboratory, corticosteroids, race, and Hispanic ethnicity, those with early disseminated disease had significantly higher WBC, neutrophils, and platelets and significantly lower hematocrit (Supplementary Table S1). The pattern and magnitude of significance were similar for those with a positive two-tier test at V1, with the exception that lymphocytes were also significantly lower (Supplementary Table S2). The proportion of patients with clinical lymphopenia was almost identical between those who were two-tier seropositive and seronegative at V1 [positive: 16/62 (25.81%) vs. negative: 44/171 (25.73%), *p* = 0.99]. The similarity of these trends is likely related to the significant association between disseminated disease and seropositivity (*n* = 233, *p* < 0.001). Similar results were also found when EM size was considered the primary predictor of interest (Supplementary Table S3), with no significant relationship between lesion size and lymphocyte count. The NLR was significantly associated with all three disease severity variables.

### Sex-based differences

**Figure 2:**
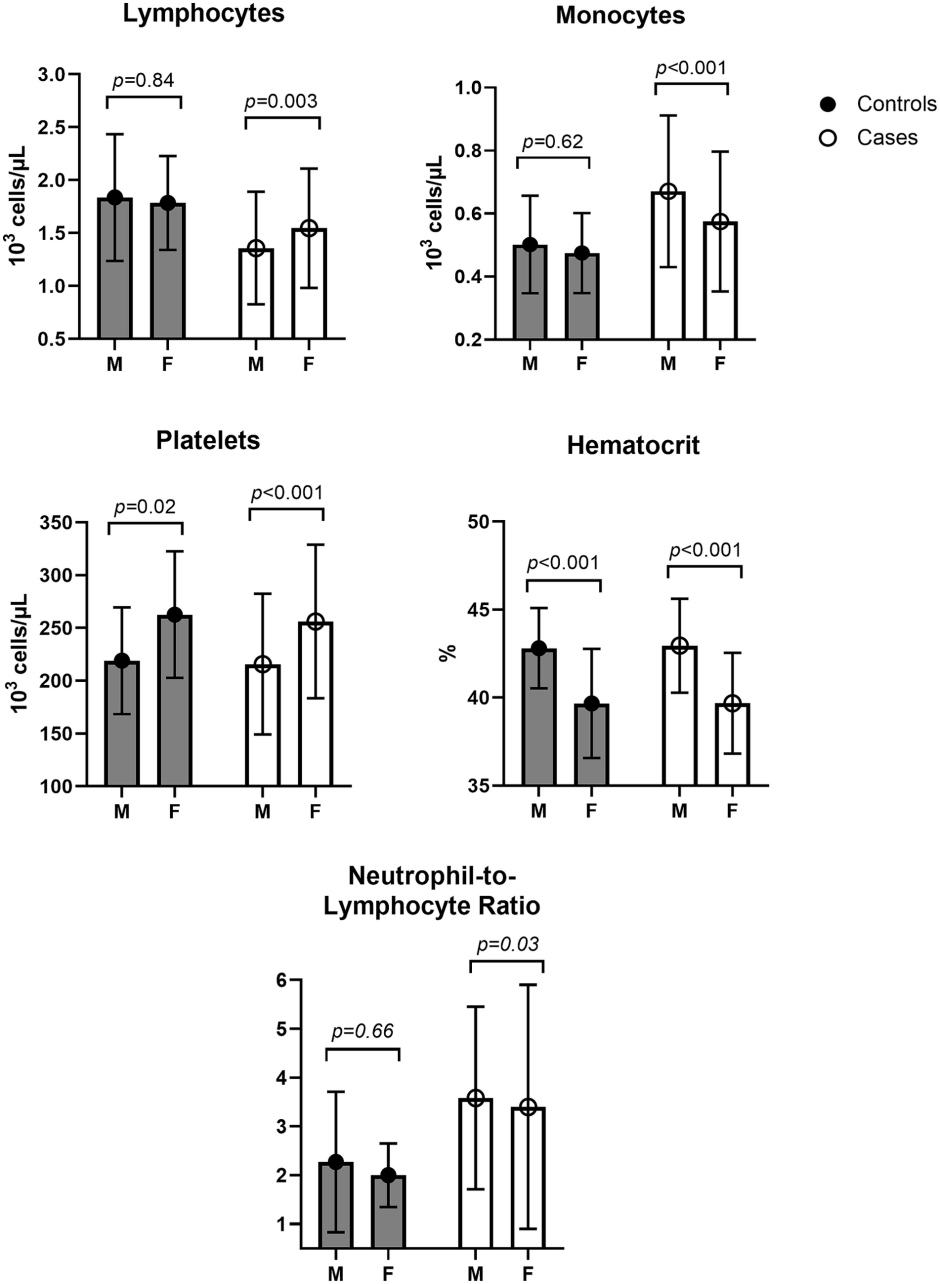
Mean and standard deviation of lymphocyte, monocyte, platelet, hematocrit, and NLR values stratified by clinical group and sex. The *p*-values represent Wilcoxon rank-sum tests comparing male and female patients within each group.

We found that sex was a significant, independent predictor of lymphocytes, monocytes, platelets, hematocrit, and NLR in adjusted models (Table 2). Therefore, we stratified our sample by group (patients with early LD and controls) and tested for sex differences in these CBC results (Figure 2). Male patients with early LD had significantly lower lymphocytes and higher monocytes than female patients with early LD, and this difference was not found among controls without LD. In contrast, known sex-based differences in platelet and hematocrit counts were consistent among participants in both groups. A higher proportion of male patients than female patients with early LD had clinical lymphopenia at V1 (31.93% vs. 19.66%, *p* = 0.03); however, there was no statistically significant difference in the proportion with monocytosis (1.68% vs. 3.42%, *p* = 0.44).

There were no significant differences by sex in the other factors independently associated with CBC results in Table 2, with the exception that male patients with early LD were younger than female patients with early LD (median 47 vs. 56 years, *p* = 0.001). When the above analyses were controlled for age, there were no significant changes to the *p*-values reported or the interpretation of their significance (data not shown). There were also no significant differences by sex in the proportion of early LD patients with disseminated disease or their duration of illness at V1.

## Discussion

In a large cohort of patients with acute, untreated LD from the Mid-Atlantic US, we found statistically significant decreases in lymphocyte counts and elevations in neutrophil and monocyte counts, both in comparison with controls without a history of LD and patients’ own pre-morbid CBC values. Given the magnitude and directionality of these changes, the strongly significant differences in the composite NLR measure that we observed would be anticipated. Moreover, the changes in lymphocyte counts we observed were more marked among male patients than female patients with early LD and were not present among controls. The association of CBC results with hours of treatment in our sample suggests rapid normalization after the initiation of appropriate antibiotic treatment.

It is important to note that among the CBC tests included in our analyses, only lymphocytes dropped such that a clinically meaningful proportion of patients (25.85%) would be considered abnormal; only 2.54 and 5.93% had abnormal monocyte and neutrophil counts, respectively. Furthermore, these changes did not result in striking alterations in the overall total WBC count. The lymphopenia and minimal neutrophilia we observed may be particularly clinically relevant in the diagnosis of patients for whom EM is not observed (15). In this setting, the differential diagnosis of non-specific, viral-like symptoms is extensive, and serologic testing is often negative at the time of initial presentation (4). A depressed lymphocyte count may be a diagnostic clue to distinguish from other acute infections such as Epstein–Barr virus, where lymphocytosis is characteristic (16). Patients with other viruses, such as acute HIV, may present with transient lymphopenia, but this may be followed by lymphocytosis (17), which has not been previously reported in LD.

Our study and others have found that acute LD rarely causes an overall elevation in the total WBC count (18). This is likely a result of mild neutrophilia in combination with lymphopenia, as observed in our study. This differs from the more prominent neutrophilia and elevated total WBC found in acute bacterial cellulitis, including streptococcal and staphylococcal infections (19, 20). The lack of an elevated WBC may provide an important diagnostic clue to consider erythema migrans in patients with atypical erythematous skin lesions. There are important therapeutic implications to differentiating between these dermatologic conditions, as many antibiotics used for cellulitis, such as first-generation cephalosporins and trimethoprim-sulfa, are not considered adequate therapy for LD (21).

Other tick-borne infections, most notably HGA, are known to cause lymphopenia as well; however, patients with HGA are often observed to also have prominent leukopenia, thrombocytopenia, and elevated transaminases (22, 23). While we did remove one patient with known HGA from our sample, we cannot be certain that co-infection with HGA was not present for a small subset of patients included in our analyses. Nevertheless, given the relative rarity of HGA in Maryland (24) and the lack of clinical suspicion in our patients, HGA is unlikely to significantly affect our findings. Moreover, prior studies have similarly reported lymphopenia in a subset of patients with EM and no evidence of concomitant HGA (25). Distinguishing between LD and HGA in the clinical setting is less relevant for treatment decisions; however, as in both cases, doxycycline is recommended.

Our findings may also be of interest in informing an understanding of the immunology of early LD. The mechanism of these acute changes observed in the CBC during *B. burgdorferi* infection is not known. *B. burgdorferi* is an extracellular pathogen without known toxin production, and selective cytotoxic loss of lymphocytes is unlikely. However, acute *B. burgdorferi* infection does induce a transient immune-mediated inflammatory response, particularly within the EM (26). Consequently, one hypothesis for a depressed lymphocyte count during acute infection could be the migration of immune cells from the blood to sites of inflammation, such as the affected skin (26).

Although we found that patients with LD’s lymphocyte and monocyte values changed relative to controls without a history of LD and their pre-morbid results, these tests were not strongly statistically significantly associated with either clinical disease dissemination or serologic positivity, two measures of initial disease severity. Relatedly, they were also not associated with estimated LD duration at V1 (lymphocytes: *r* = 0.04, *p* = 0.53; monocytes: *r* = −0.08, *p* = 0.22). This suggests that these changes can occur early in infection and may be clinically present prior to the development of detectable antibodies, as evidenced by the lack of association with two-tier antibody status. Indeed, among tests found to differ between patients with early LD and controls, only neutrophil counts and the NLR were clearly associated with these clinical factors. Notably, the composite NLR was strongly statistically significantly associated with disease severity even when individual lymphocyte or neutrophil counts were not.

We observed that demographic factors, such as sex, age, and race, were frequently independently associated with specific CBC results. Prior studies have noted that such factors may affect CBC results and interpretation among healthy individuals (9–11), although this may not be widely appreciated by clinical practitioners. We also found that changes in the acute lymphocyte count in early LD, including the proportion of patients with clinical lymphopenia, were more pronounced among male than female patients in our sample. It has been shown that female patients generally mount a stronger innate immune response to infection than male patients (27); however, the immunologic basis for our findings is not currently known as sex differences in response to infection with *B. burgdorferi* have not been comprehensively characterized. Regardless, the use of lymphopenia as a diagnostic clue may be more applicable to male patients, an important caveat for future practice guidelines. In addition, these findings reinforce that studies seeking to understand acute *B. burgdorferi* infection should ensure sex disaggregation of data to fully and equitably characterize the host immunologic response. While we chose to use the term “sex” in this manuscript based on the assumption that any differences we observed are more likely to result from biological determinants, it is ultimately difficult to discern the relevance of social factors as well.

As previously noted, our study is limited by the lack of HGA testing in all patients to confirm the absence of tick-borne co-infections in our sample. Similarly, while all patients with LD were diagnosed clinically with EM present, their disease was not confirmed through direct detection of *B. burgdorferi*. As a result, the presence of other conditions such as Southern tick-associated rash illness (STARI) (28) could not be identified. Furthermore, it would have been preferable if the LD patient and control groups had more comparable sample sizes as this may have affected our ability to detect group differences. As noted, the translation of our findings to clinical practice should account for the fact that the magnitude of the differences in CBC tests that we observed was relatively modest. In addition, it would have been preferable if all CBC tests were performed by the same laboratory. Nevertheless, we controlled for laboratory in adjusted analyses, and it did not appear to significantly affect our findings. Finally, given how our data were recorded, it is a limitation that we cannot be certain that we accurately captured biological sex for a small proportion of patients. However, the proportion of participants with known transgender and/or non-binary status in our sample (0.46%) is very similar to estimates of the US population (29, 30), and we do not expect this to have significantly biased our findings.

Until there is broad clinical availability of tests to more accurately and rapidly identify patients with early LD, practitioners must continue to also rely on clinical history, physical examination, and other available tools to inform diagnosis. In the appropriate geographic and seasonal context, the presence of lymphopenia and the absence of an elevated total WBC, even among two-tier seronegative patients, make the diagnosis of LD an important consideration. Importantly, this may be especially true for men, and future studies are needed to clarify sex-based differences in both the clinical presentation and immunologic response to *B. burgdorferi* infection. Although an elevated NLR appears to offer a promising clue in the diagnosis of early LD based on our findings, future studies are also needed to further understand its relevance and refine appropriate disease cutoffs.

## Data Availability

The raw data supporting the conclusions of this article will be made available by the authors, without undue reservation.
