# Supplementary material for: A sex-based analysis of complete blood count features during acute, untreated Lyme disease
Source: Front Med (Lausanne). 2024 Oct 28;11:1454858. doi: 10.3389/fmed.2024.1454858 (PMC11551033; doi:10.3389/fmed.2024.1454858)
Supplement: Supplementary file 1 [file Data_Sheet_1.docx]

**Supplemental Table 1**. Adjusted linear regression models among antibiotic naïve patients with early Lyme disease to predict CBC test result values, with disseminated disease as the primary independent variable of interest and the following included as potential confounders: age, sex, laboratory, race (Black vs. other and White vs. other), Hispanic ethnicity, and corticosteroids. Only variables identified as significant (p<0.15) in univariate analyses were included in a final adjusted model and presented in the table below.

|  |  | **Unadjusted** | | **Adjusted** | |
| --- | --- | --- | --- | --- | --- |
| **Outcome** | **Predictors** | **Co-efficient (95% CI)** | **p-value** | **Co-efficient (95% CI)** | **p-value** |
| White Blood Cell Count | Disseminated | 0.71 [0.19, 1.22] | 0.008 | 0.71 [0.19, 1.22] | 0.008 |
| Lymphocyte Count | Disseminated | -0.12 [-0.26, 0.02] | 0.08 | -0.11 [-0.25, 0.03] | 0.13 |
|  | Female | 0.20 [0.08, 0.33] | 0.002 | 0.20 [0.07, 0.33] | 0.003 |
| Neutrophil Count | Disseminated | 0.81 [0.39, 1.24] | < 0.001 | 0.79 [ 0.37, 1.21] | < 0.001 |
|  | Laboratory | 0.61 [-0.03, 1.26] | 0.06 | 0.54 [-0.10, 1.18] | 0.10 |
| Monocyte Count | Disseminated | -0.02 [-0.07, 0.04] | 0.60 | -0.02 [-0.08, 0.04] | 0.50 |
|  | Age (10 years) | -0.02 [-0.04, 0.00] | 0.05 | -0.01 [-0.03, 0.01] | 0.15 |
|  | Female | -0.10 [-0.15, -0.04] | < 0.001 | -0.09 [-0.15, -0.04] | 0.001 |
|  | Laboratory | 0.07 [-0.02, 0.16] | 0.11 | 0.07 [-0.02, 0.16] | 0.14 |
|  | Corticosteroids | 0.20 [0.03, 0.37] | 0.02 | 0.21 [0.04, 0.38] | 0.01 |
| Platelet Count | Disseminated | 33.35 [14.45, 52.24] | 0.001 | 36.32 [17.07, 55.58] | < 0.001 |
|  | Female | 39.74 [22.23, 57.25] | < 0.001 | 41.47 [23.74, 59.21] | < 0.001 |
| Hematocrit Count | Disseminated | -1.03 [-1.96, -0.10] | 0.03 | -1.43 [-2.23, -0.62] | < 0.001 |
|  | Age (10 years) | -0.30 [-0.58, -0.02] | 0.04 | 0.02 [-0.24, 0.27] | 0.89 |
|  | Female | -3.24 [-3.96, -2.51] | < 0.001 | -3.40 [-4.16, -2.65] | < 0.001 |
| Neutrophil-to-Lymphocyte Ratio | Disseminated  Female  Laboratory | 0.93 [0.49, 1.36]  -0.39 [-0.81, 0.03]  0.74 [0.05, 1.44] | < 0.001  0.07  0.04 | 0.86 [0.43, 1.30]  -0.30 [-0.70, 0.11]  0.62 [-0.04, 1.29] | < 0.001  0.15  0.07 |

**Supplemental Table 2**. Adjusted linear regression models among antibiotic naïve patients with early Lyme disease to predict CBC test result values, with two-tier serology result as the primary independent variable of interest and the following included as potential confounders: age, sex, laboratory, race (Black vs. other and White vs. other), Hispanic ethnicity, and corticosteroids. Only variables identified as significant (p<0.15) in univariate analyses were included in a final adjusted model and presented in the table below.

|  |  | **Unadjusted** | | **Adjusted** | |
| --- | --- | --- | --- | --- | --- |
| **Outcome** | **Predictors** | **Co-efficient (95% CI)** | **p-value** | **Co-efficient (95% CI)** | **p-value** |
| White Blood Cell Count | Seropositive | 1.04 [0.50, 1.58] | < 0.001 | 1.04 [0.50, 1.58] | < 0.001 |
| Lymphocyte Count | Seropositive | -0.15 [-0.30, 0.00] | 0.05 | -0.14 [-0.29, 0.00] | 0.05 |
|  | Female | 0.20 [0.08, 0.33] | 0.002 | 0.21 [0.09, 0.34] | 0.001 |
| Neutrophil Count | Seropositive | 1.25 [0.82, 1.68] | < 0.001 | 1.21 [ 0.78, 1.64] | < 0.001 |
|  | Laboratory | 0.61 [-0.03, 1.26] | 0.06 | 0.38 [-0.25, 1.01] | 0.24 |
| Monocyte Count | Seropositive | 0.001 [-0.06, 0.06] | 0.96 | -0.002 [-0.06, 0.06] | 0.92 |
|  | Age (10 years) | -0.02 [-0.04, 0.00] | 0.05 | -0.01 [-0.03, 0.01] | 0.13 |
|  | Female | -0.10 [-0.15, -0.04] | < 0.001 | -0.09 [-0.14, -0.03] | 0.002 |
|  | Laboratory | 0.07 [-0.02, 0.16] | 0.11 | 0.07 [-0.02, 0.16] | 0.14 |
|  | Corticosteroids | 0.20 [0.03, 0.37] | 0.02 | 0.21 [0.04, 0.38] | 0.01 |
| Platelet Count | Seropositive | 42.55 [22.65, 62.45] | < 0.001 | 45.36 [26.02, 64.69] | < 0.001 |
|  | Female | 39.74 [22.23, 57.25] | < 0.001 | 41.88 [24.79, 58.97] | < 0.001 |
| Hematocrit Count | Seropositive | -1.81 [-2.79, -0.83] | < 0.001 | -2.02 [-2.83, -1.21] | < 0.001 |
|  | Age (10 years) | -0.30 [-0.58, -0.02] | 0.04 | 0.01 [-0.23, 0.26] | 0.91 |
|  | Female | -3.24 [-3.96, -2.51] | < 0.001 | -3.34 [-4.07, -2.61] | < 0.001 |
| Neutrophil-to-Lymphocyte Ratio | Seropositive  Female  Laboratory | 1.28 [0.83, 1.73]  -0.39 [-0.81, 0.03]  0.74 [0.05, 1.44] | < 0.001  0.07  0.04 | 1.21[ 0.77, 1.66]  -0.38 [-0.77, 0.01]  0.51 [-0.14, 1.16] | < 0.001  0.06  0.12 |

**Supplemental Table 3**. Adjusted linear regression models among antibiotic naïve patients with early Lyme disease to predict CBC test result values, with erythema migrans lesion size (in cm^2^, scaled by dividing by 100) as the primary independent variable of interest and the following included as potential confounders: age, sex, laboratory, race (Black vs. other and White vs. other), Hispanic ethnicity, and corticosteroids. Only variables identified as significant (p<0.15) in univariate analyses were included in a final adjusted model and presented in the table below.

|  |  | **Unadjusted** | | **Adjusted** | |
| --- | --- | --- | --- | --- | --- |
| **Outcome** | **Predictors** | **Co-efficient (95% CI)** | **p-value** | **Co-efficient (95% CI)** | **p-value** |
| White Blood Cell Count | Rash Size | 0.22 [0.08, 0.37] | 0.002 | 0.22 [0.08, 0.37] | 0.002 |
| Lymphocyte Count | Rash Size | -0.01 [-0.05, 0.03] | 0.60 | 0.002 [-0.04, 0.04] | 0.94 |
|  | Female | 0.20 [0.08, 0.33] | 0.002 | 0.21 [ 0.08, 0.34] | 0.002 |
| Neutrophil Count | Rash Size | 0.24 [0.12, 0.35] | < 0.001 | 0.23 [ 0.11, 0.34] | < 0.001 |
|  | Laboratory | 0.61 [-0.03, 1.26] | 0.06 | 0.59 [-0.04, 1.22] | 0.07 |
| Monocyte Count | Rash Size | 0.03 [0.01, 0.04] | < 0.001 | 0.02 [ 0.01, 0.04] | 0.01 |
|  | Age (10 years) | -0.02 [-0.04, 0.00] | 0.05 | -0.01 [-0.03, 0.00] | 0.11 |
|  | Female | -0.10 [-0.15, -0.04] | < 0.001 | -0.07 [-0.13, -0.02] | 0.007 |
|  | Laboratory | 0.07 [-0.02, 0.16] | 0.11 | 0.07 [-0.02, 0.15] | 0.14 |
|  | Corticosteroids | 0.20 [0.03, 0.37] | 0.02 | 0.26 [0.08, 0.44] | 0.005 |
| Platelet Count | Rash Size | 4.29 [-1.07, 9.65] | 0.12 | 7.48 [2.13, 12.84] | 0.006 |
|  | Female | 39.74 [22.23, 57.25] | < 0.001 | 45.86 [27.92, 63.80] | < 0.001 |
| Hematocrit Count | Rash Size | 0.23 [-0.02, 0.49] | 0.07 | 0.004 [-0.22, 0.23] | 0.97 |
|  | Age (10 years) | -0.30 [-0.58, -0.02] | 0.04 | -0.03 [-0.28, 0.22] | 0.83 |
|  | Female | -3.24 [-3.96, -2.51] | < 0.001 | -3.17 [-3.93, -2.42] | < 0.001 |
| Neutrophil-to-Lymphocyte Ratio | Rash Size  Female  Laboratory | 0.21 [0.09, 0.33]  -0.39 [-0.81, 0.03]  0.74 [0.05, 1.44] | < 0.001  0.07  0.04 | 0.18 [ 0.06, 0.30]  -0.26 [-0.67, 0.14]  0.67 [0.02, 1.32] | 0.004  0.20  0.04 |
